# Effects of Varying Ratios of *Glycyrrhiza uralensis* and Donkey Hide Gelatin Water Extracts on Dinitrochlorobenzene-Induced Atopic Dermatitis in NC/Nga Mice

**DOI:** 10.3390/nu15092094

**Published:** 2023-04-26

**Authors:** Linsha Dong, Ju Hyun Lee, Eun Heui Jo, Jin-Sil Lee, Seung-Hyung Kim, Dong-Sung Lee, Min Cheol Park

**Affiliations:** 1 College of Pharmacy, Chosun University, Dong-gu, Gwangju 61452, Republic of Korea; donglinsha011@163.com; 2 Department of Ophthalmology and Otolaryngology and Dermatology, Wonkwang University Korean Medicine Hospital and Research Center of Traditional Korean Medicine, Wonkwang University, Iksan 54538, Republic of Korea; tony9403@naver.com; 3 Department of Acupuncture and Moxibustion, Wonkwang University Korean Medicine Hospital and Research Center of Traditional Korean Medicine, Wonkwang University, Iksan 54538, Republic of Korea; freezo@wonkwang.ac.kr; 4 Director of R & D Institute, Haewon Biotech Inc., Yongin 59143, Republic of Korea; jsleefn@naver.com; 5 Institute of Traditional Medicine and Bioscience Daejeon University, Daejeon 34520, Republic of Korea; sksh518@dju.kr

**Keywords:** atopic dermatitis, donkey hide gelatin, *Glycyrrhiza uralensis*, HaCaT cell, RAW264.7 cell

## Abstract

Atopic dermatitis is a chronic skin disease that affects millions of people all over the world. The objective of this study was to evaluate the inhibitory effects of the roots of *Glycyrrhiza uralensis* (GU) and Donkey Hide Gelatin (DHG) water extracts on DNCB-induced NC/Nga mice and TNF-α/IFN-γ treated keratinocytes or LPS-stimulated macrophages. The combined treatment using the water extracts of GU and DHG improved the skin symptom evaluation score and skin histology, with increased expression of the skin barrier proteins Claudin 1 and Sirt 1 in lesion areas. The IFN-γ activity was promoted in PBMCs, ALN, and dorsal skin tissue, while the absolute cell number was reduced for T cells so that the production and expression of serum IgE and cytokines were suppressed. In TNF-α/IFN-γ induced HaCaT cells, IL-6, IL-8, MDC, and RANTES were all inhibited by GU and DHG water extracts, while ICAM-1 and COX-2 levels were similarly downregulated. In addition, GU and DHG water extracts decreased LPS-mediated nitric oxide, IL-6, TNF-α, and PGE_2_ in RAW 264.7 cells, and the expression of iNOS and COX-2 also decreased. Notably, the DHG:GU ratio of 4:1 was shown to have the best effects of all ratios. In conclusion, GU and DHG have anti-skin inflammatory potentials that can be used as alternative ingredients in the formula of functional foods for people with atopic dermatitis.

## 1. Introduction

Inflammatory skin lesions are the hallmark of the chronic, recurring skin condition known as atopic dermatitis (AD) [1]. Its precise pathological mechanism remains to be elucidated, although it is generally known that skin barrier damage and immunological abnormalities are the major factors contributing to the further development of atopic dermatitis [2]. The skin barrier is composed of tight junctions (TJ). TJs are connection structures between cells that exist in simple, multi-layered epithelia and endothelia and consist of transmembrane proteins (claudins, junctional adhesion molecule A (JAM-A), TJ-associated marvel proteins (TAMP) (occludin and tricellulin), and TJ plaque proteins (the zonula occludens proteins ZO-1 and ZO-2, MUPP-1, cingulin, and symplekin) [3]. It acts as a kind of barrier against the invasion of external allergens on the surface of the human body’s skin. Damage to the skin barrier leads to a decline in the defense activity of the skin, thus facilitating the invasion of allergens and other external factors, with increased skin cell damage and sensitization [4,5]. AD affects up to 20% of children and up to 3% of adults, and the rate of incidence is steadily increasing with time, especially in low-income countries [5]. The increased incidence of atopic dermatitis has led to increased socio-economic costs for its treatment and management, which, in turn, has gradually increased current interest in novel therapeutic agents [6,7].

An important aspect of AD is the production and secretion of pro-inflammatory cytokines and chemokines. During acute and chronic stages of AD [8], elevated secretion of cytokines aggravates the infiltration of immune and inflammatory cells into inflammatory skin lesions, such as mast cells, eosinophils, and T lymphocytes [9]. Macrophages have an essential role in the production of pro-inflammatory mediators. Nitrite and PGE_2_ have been reported to be significant in a number of physiological processes, such as vasodilation, neurotransmission, blood coagulation, and immunological modulation, among the numerous pro-inflammatory mediators [10,11]. A Th1/Th2 imbalance caused by chemokines can result in AD lesions. Chemokines are produced by keratinocytes and are important in the development of inflammatory skin illnesses [11,12].

The drug efficacy varies according to the type, concentration, and mixing ratio of drug components [13,14,15], which makes it essential that the type, dose, and proportion of the drugs be adjusted to suitable levels prior to their administration so as to enhance the original drug effects [16]. The current focus of many studies is on the additive effect of the drugs when administered concomitantly as the most efficient method to maximize the drug’s efficacy. Recent studies have shown that the combination of several different compounds exhibits a synergistic effect to produce various therapeutic effects, such as analgesic and anti-viral effects [17].

Several studies have reported that the roots of *Glycyrrhiza uralensis* (GU) and donkey hide gelatin (DHG), which are used in health foods and therapeutic materials, have anti-allergic and anti-inflammatory properties. NC/Nga mice have atopic susceptibility traits, and their skin lesions and pathological and immunological characteristics are consistent with human atopic dermatitis. In recent years, research has been conducted on the genetics, pathogenesis, and traditional Chinese and Western medicine treatment of atopic dermatitis using NC/Nga mice model [18]. However, no study has demonstrated the anti-atopic efficacy of these agents combined as GU-DHG extract yet. Based on the findings of previous studies that support the anti-inflammatory and anti-allergic effects of GU and DHG, this study was designed to verify the inhibitory effects of GU and DHG water extracts and varying proportions of the two drugs on dinitrochlorobenzene (DNCB)-induced AD in NC/Nga mice. We also investigated the anti-AD activity of GU and DHG water extracts in human keratinocytes (HaCaT) as well as macrophages (RAW 264.7), which had been activated with TNF-α/IFN-γ and LPS, respectively.

## 2. Materials and Methods

### 2.1. Animal

The 7-week-old male NC/Nga mice (Charles River Laboratories, Shizuoka, Japan) used in this study were obtained from OrientBio, Inc. (Seongnam, Republic of Korea). The mice were kept in a general breeding room for one week of acclimatization prior to the experiment. All experiments in this study were conducted with the approval of the Institutional Review Board and Animal Experiment Ethics Committee of Daejeon University (IRB Approval No.: DJUARB2019-041).

### 2.2. Atopic Dermatitis Induced by DNCB

After acclimatization, hair removal from the base of the ear to the tip of the tail, and a 24-h rest period. After that, the shaved region received 200 μL of 1% DNCB dissolved in acetone and olive oil (3:1), which was then applied again three days later. An amount of 150 μL of 0.4% DNCB was administered three times per week for five weeks, starting on the seventh day following the first DNCB application.

### 2.3. Sample Preparation

The GU and DHG used in this study were purchased from Wonchang Pharma (Cheonan, Republic of Korea) and CK Pharma Co. (Seoul, Republic of Korea). A voucher specimen of DHG (GA023) was stored at Wonchang Pharma, and that of GU (G143-1-504) was managed by CK Pharma Co. For the experiment, 1040 mL of purified water and 30 g of DHG and GU each were added, and the extraction was done using a decoction device (KS-220 (25 L), Kyungseo E&P, Incheon, Republic of Korea). Using a vacuum distillation unit, the filtrate was enhanced, and the enriched filtrate was thoroughly dried in a freeze dryer before being kept in a freezer (−80 °C) for later use. With this procedure, 30 g of DHG and 30 g of GU were separated into 17.63 g and 5.65 g, respectively, of a freeze-dried powder extract.

### 2.4. Intervention

A total of 60 male NC/Nga mice were randomly separated into 10 groups (*n* = 6); groups are shown in Table 1. The experimental groups, consisting of mice with DNCB-induced atopic dermatitis, were orally administered 0.2 mL of the following at a fixed time each day for 3 weeks: 100 mg/kg GU, 100 mg/kg DHG, 200 mg/kg GU:DHG = 1:1, 200 mg/kg GU:DHG = 1:2, 200 mg/kg GU:DHG = 1:4, 200 mg/kg GU:DHG = 2:1, 200 mg/kg GU:DHG = 4:1, and 3 mg/kg dexamethasone administered to DNCB_GU 100 mg/kg, DNCB_DHG 100 mg/kg, DNCB_GU1:DHG1 200 mg/kg, DNCB_GU1:DHG2 200 mg/kg, DNCB_GU1:DHG4 200 mg/kg, DNCB_GU2:DHG1 200 mg/kg, DNCB_GU4:DHG1 200 mg/kg, and DNCB_Dexa, respectively. To collect skin tissues and blood serum, the experimental animals were euthanized by cervical dislocation after being given ether anesthesia.

### 2.5. Evaluation of Atopic Dermatitis Skin Symptoms

Seven days following the application of DNCB, the skin symptom evaluation score was assessed every seven days. NC/Nga mice with stimulated atopic dermatitis were evaluated for the following symptoms: (1) erythema/hemorrhage; (2) scarring/dryness; and (3) excoriation/erosion. The symptoms were recorded as a score of 0 (none), 1 (mild), 2 (moderate), and 3 (severe); the sum of these scores was used to determine the mice’s atopic dermatitis score.

### 2.6. Analysis of Dorsal Skin Tissue, Axillary Lymph Node (ALN), and Peripheral Blood Mononuclear Cells (PBMCs) Using Fluorescence-Activated Cell Sorting

The total cell count was calculated from the dorsal skin and separated ALN tissues. Then, by adjusting the tissue cell number to 5 × 10^5^, immunofluorescence staining was performed for all tissues at 4 °C. Each tissue was incubated for 30 min on ice with anti-CD3e-PE, anti-CD4-FITC, anti-CD8-FITC, anti-CD11b-FITC, anti-CD23-FITC, anti-ti-CD69-FITC, anti-Gr-1 FITC, anti-CCR3-PE, and anti-B220-PE. The tissues were then rinsed with phosphate-buffered saline (PBS) at least three times. The flow cytometry’s Cell Quest tool was used to assess the cell count as a percentage (%); the total cell count was then used to infer the precise number of cells in each tissue.

### 2.7. Immunoglobulin E (IgE) Level Measurement

Each mouse had 100 mL of blood drawn at the end of the experiment, which was centrifuged for 20 min at 6500 rpm to separate 30 mL of serum. Of this, 5 μL of the isolated serum was mixed with a 45 μL dilution buffer, and the mixture was aliquoted to each well and incubated at 25 °C for 2 h, followed by washing twice. Next, conjugated anti-biotin antibody-IgE was added and left to stand for 2 h. The contents of the well were washed twice with water and washing buffer. Next, 100 μL conjugated anti-avidin antibody-horseradish peroxide (HRP) was added for 1 h, followed by washing. Lastly, 100 μL of TMB substrate was aliquoted for 30 min, and after adding the stop solution, the OD value was measured at 450 nm using an enzyme-linked immunoassay (ELISA) reader.

### 2.8. Detection of Th1/Th2 Cytokine Expression

At the end of the experiment, the spleen of each mouse was extracted, and splenocytes were isolated using a 100-mesh sieve. Using the ammonium-chloride-potassium (ACK) solution, the red blood cells (RBC) were removed from the isolated splenocytes, and the treated splenocytes were aliquoted into wells coated with DNCB (5 × 10^5^ cells/well) and cultured for 48 h. The resulting culture solution was centrifuged at 2000 rpm for 3 min, and through this process, 200 μL of culture supernatant was obtained from the splenocytes. The levels of IL-4, IFN-gamma, IL-5 (BioSource, San Diego, CA, USA), and IL-13 (R&D Systems, Minneapolis, MN, USA) in the supernatant were measured by ELISA.

### 2.9. Quantitative Polymerase Chain Reaction (qPCR) in Dorsal Skin Tissue

For cytokine gene expression, SYBR® Green PCR Master (ABI, San Diego, CA, USA) Mix was used, and for the internal standard GAPDH, TaqMan® probe (ABI, San Diego, CA, USA) was used. The reaction was continued until the final concentration of the primer was 200 nM. The mRNA expression of IL-31R, IL-13, COX-2, and TNF-α in dorsal skin tissue was analyzed using DNA synthesis. The qPCR cycling conditions were as follows: 0.15 min at 95 °C and 1 min at 60 °C for 40 cycles, with a pre-denaturation condition of 2 min at 50 °C and 10 min at 94 °C. The base sequences of the mouse oligonucleotides are as shown below (Table 2).

### 2.10. Histology Analysis

To examine the condition of the skin tissue, the paraffin-formatted fixed tissue was sectioned into a block with a thickness of 5 μm. On the block, toluidine blue and hematoxylin/eosin (H&E) stains were used under a light microscope (×200, Nikon, Tokyo, Japan).

### 2.11. Immunofluorescence to Measure Claudin 1 and Sirtuin 1 (Sirt 1) Expression in the Epidermis

The primary antibody was diluted with blocking buffer for 4 h and washed 3 times. The secondary antibody was also diluted with blocking buffer for 2 h in a shaded area and washed with PBST for 10 min. To stain the nuclei, the cells were washed for 10 min with PBST that had previously been reacted with Hoechst 33258, mounted using Gel Mount™ and a cover glass, and placed for drying in a dark room at room temperature. Next, the cells were incubated with the primary antibody (Claudin 1, ab15098; Sirt 1, ab189494; and Hoechst 33258, ab228550; Abcam, Cambridge, MA, USA), then incubated with the secondary antibody (ab205718; Abcam, MA, USA) for anti-rabbit FITC-conjugated IgG. The fluorescent microscope (Zeiss LSM 510, Carl Zeiss, Oberkochen, Germany) was used to examine the changes in Claudin 1 and Sirt 1 expression in the epidermis.

### 2.12. Cell Culture and MTT Assay

To determine cell viability, HaCaT cells and RAW264.7 cells were treated with extract samples: DHG, GU1:DHG4, GU1:DHG2, GU1:DHG1, GU2:DHG1, GU4:DHG1, and GU. The concentration of every sample is 50–200 μg/mL. For the experimental steps of MTT, please refer to our previous study [19].

### 2.13. Measurement of Nitric Oxide and Cytokines in RAW264.7 Cells

The RAW264.7 cells were pretreated for 3 h with 1–7 samples, as shown in Table 3, and then treated with 1 μg/mL LPS for 24 h. After that, the cell culture supernatant was collected for further experimentation and the detection of nitric oxide, IL-6, TNF-α, and PGE_2_ secretions. Nitric oxides were detected by Griess reagent. IL-6, TNF-α and PGE_2_ secretions were evaluated using specific ELISA kits, following the instructions of the manufacturer.

### 2.14. Measurement of Chemokines and Cytokines in HaCaT Cells

The cell supernatant was collected for further experimentation and detection of IL-6, IL-8, MDC, and RANTES secretions. They were evaluated with specific ELISA kits, following the instructions of the manufacturer.

### 2.15. NF-κB Binding Activity

NF-κB p65-DNA binding was detected using an NF-κB p65 Transcription Factor Assay Kit (10007889, Cayman Chemical). All steps were according to the manufacturer’s instructions.

### 2.16. Isolation of Nuclear and Cytoplasmic Fractions

HaCaT cells and RAW264.7 cells were pre-treated for 3 h with DHG 200 μg/mL (1), GU1:DHG4 100 μg/mL (2), GU1:DHG2 100 μg/mL (3), GU1:DHG1 100 μg/mL (4), GU2:DHG1 100 μg/mL (5), GU4:DHG1 100 μg/mL (6), and GU 100 μg/mL (7), and then stimulated for 15 min with TNF-α/IFN-γ (10 ng/mL) or LPS (1μg/mL). After stimulation, the cells were scraped and harvested. Nuclear and cytoplasmic protein fractions were extracted following the Extraction Reagents Kit (Caymen, Ann Arbor, MI, USA), following the manufacturer’s instructions.

### 2.17. Western Blot Analysis

Pre-treated with 1–7 samples for 3 h, then HaCaT cells were induced with TNF-α/IFN-γ (10 ng/mL) for 24 h. RAW264.7 cells were induced with LPS (1 μg/mL) for 24 h. After being scraped and extracted, the cells were lysed using radioimmunoprecipitation assay (RIPA) buffer. SDS-PAGE gel was used to separate the proteins, and the membranes used were NC membranes. Using 5% skim milk, the membrane was blocked for one hour. Afterwards, primary antibodies were incubated for a further day at 4 °C. After being washed with TBST (Tris-buffer with Tween-20), the membranes were incubated with an HRP-conjugated secondary antibody for 60 min. ECL solution was used to identify certain proteins after TBST washing. The ImageJ program was used to evaluate the membranes (NIH, Rockville, MD, USA).

### 2.18. UHPLC Analysis

For the methods of UHPLC analysis of the GU1:DHG4 extract, please refer to our previous article [19].

### 2.19. Statistical Analysis

The numerical data of the experimental groups were expressed as mean ± standard deviation (SD). Cell assays were evaluated using an independent sample t-test, while animal experiments were evaluated using one-way analysis of variance (ANOVA). To test the significance of the results, Duncan’s multiple comparison test was performed. The cases of *p* < 0.05, 0.01, or 0.001 were differentiated for the analysis (^#^ *p* < 0.05, ^##^ *p* < 0.01, ^###^ *p* < 0.001 vs. NC/Nga_Nr; ** *p* < 0.01 and *** *p* < 0.001 vs. DNCB-CTL.) to verify the statistical significance.

## 3. Results

### 3.1. Changes in Body Weight

**Figure 1:**
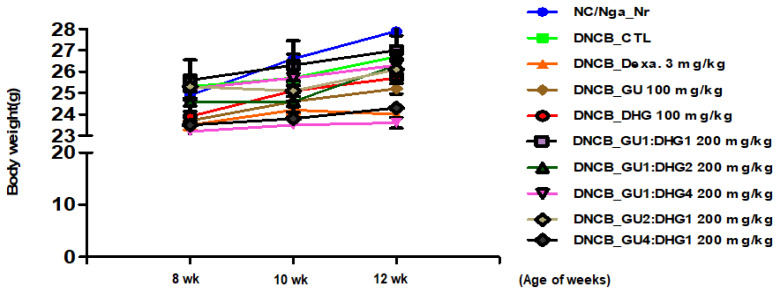
Changes in the body weight of experimental mice.

To check the changes in body weight. The results of the body weight of the mice show that most groups have a slight weight gain, but there is no statistical difference in weight between different groups (*p* < 0.05) (Figure 1).

### 3.2. Changes in Skin Symptom Evaluation Score

**Figure 2:**
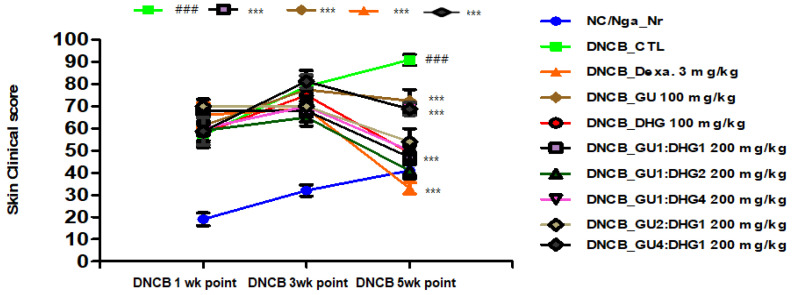
Clinical skin features and severity of atopic dermatitis skin lesions in NC/Nga mice with DNCB-induced atopic dermatitis (*n* = 6). ^###^ *p* < 0.001 compared with NC/Nga_Nr, *** *p* < 0.001 compared with DNCB-CTL.

**Figure 3:**
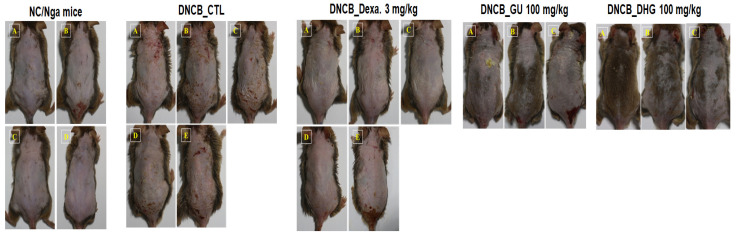

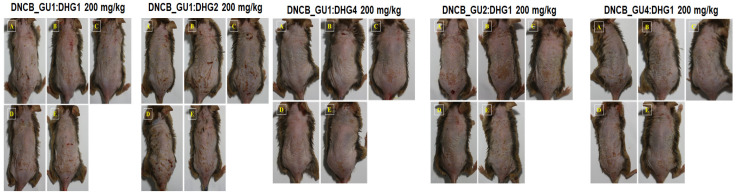
Topical application of dexamethasone, GU, DHG, and GU + DHG mixtures of clinical features of dermatitis in NC/Nga mice (*n* = 6) (A–E is a representative image of 4 to 5 mice).

The score of skin symptom evaluation for DNCB_CTL showed ≥4-fold increase, which was a significant change compared to NC/Nga_Nr, which was free of DNCB application. On the contrary, the score of skin symptom evaluation in DNCB_Dexa and the experimental groups indicated a noticeable decline compared to DNCB_CTL, in the following order for the experimental groups: DNCB_DHG 100 mg/kg, DNCB_GU 100 mg/kg, DNCB_GU4:DHG1 200 mg/kg, DNCB_GU2:DHG1 200 mg/kg, DNCB_GU1:DHG1 200 mg/kg, DNCB_GU1:DHG2 200 mg/kg, and DNCB_GU1:DHG4 200 mg/kg, with DNCB_DHG 100 mg/kg, DNCB_GU1:DHG4 200 mg/kg, DNCB_GU 100 mg/kg, and DNCB_GU1:DHG1 200 mg/kg, showed the significant decrease (Figure 2 and Figure 3).

### 3.3. Changes in CD4+/CD69+ and Gr-1+/CD11b+ Cell Frequency through PBMC Immunofluorescence Staining

**Figure 4:**
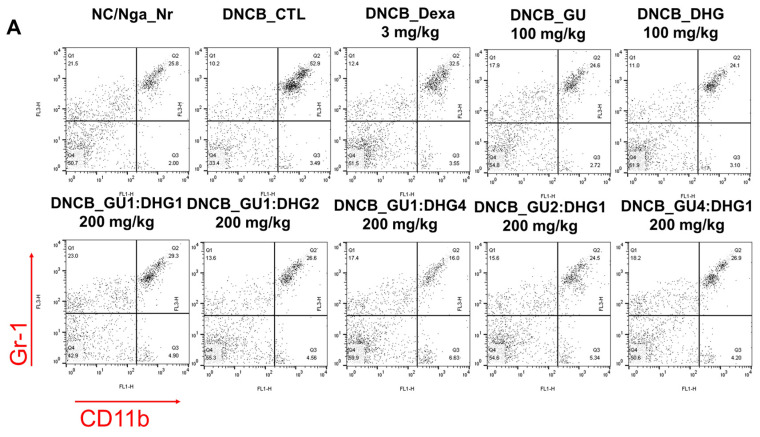

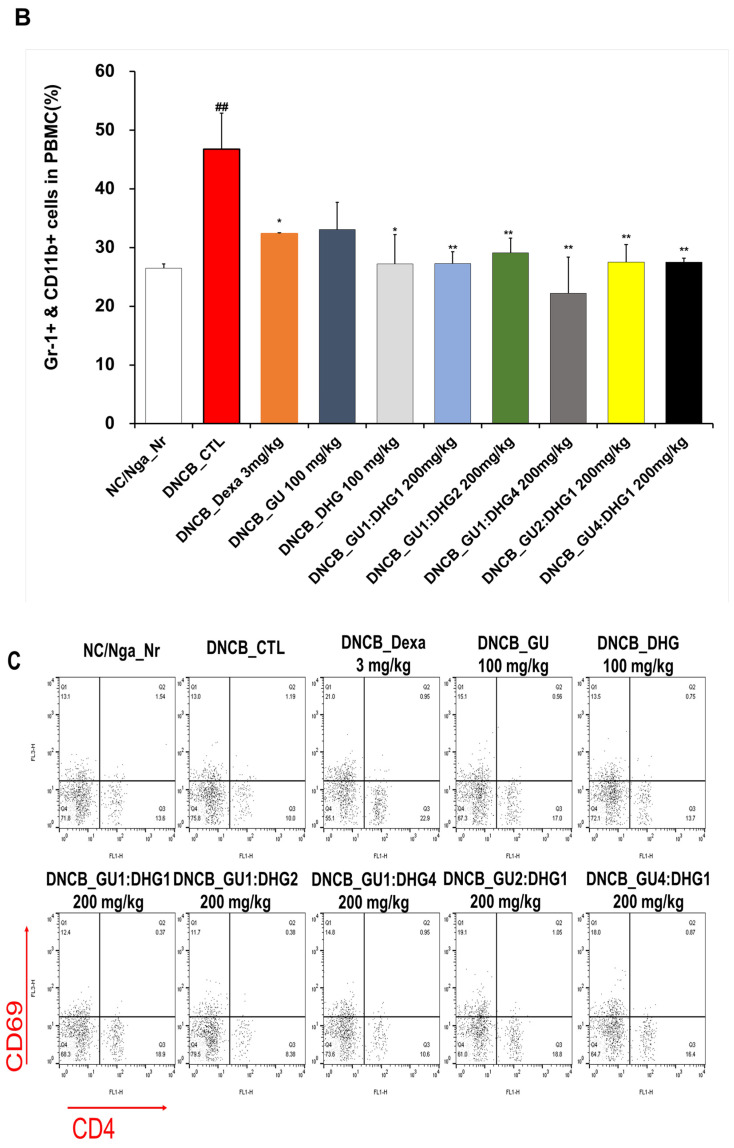

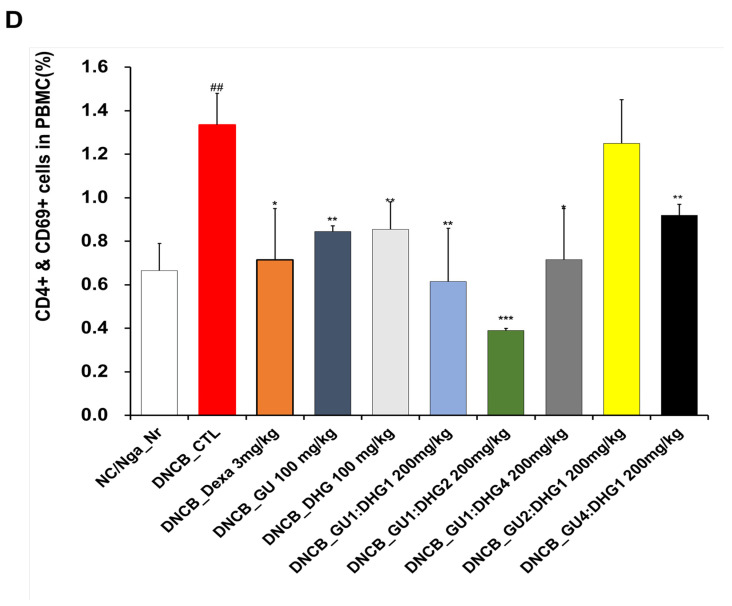
Effects of dexamethasone, GU, DHG, and GU + DHG mixtures on the percentage of CD4+/CD69+ and Gr-1+/CD11b+ changes in total cell content numbers in PBMCs in NC/Nga mice by DNCB (*n* = 6). (**A**–**D**) ^##^ *p* < 0.01 compared with NC/Nga_Nr, * *p* < 0.05, ** *p* < 0.01, *** *p* < 0.01 compared with DNCB-CTL.

The FACS-based PBMC analysis showed that the total cell frequencies for CD4+/CD69+ and Gr-1+/CD11b+ in DNCB_CTL were significantly increased compared to those in NC/Nga_Nr. The total cell frequency of CD4+/CD69+ was significantly lower than DNCB_CTL across all experimental groups except DNCB_GU2:DHG1 200 mg/kg, while the rate of decrease was the highest for DNCB_GU1:DHG2 200 mg/kg. The total cell frequency of Gr-1+/CD11b+ was also significantly lower in DNCB_Dexa and across all experimental groups that received the combined treatment with varying ratios, with the most notable change observed for DNCB_GU1:DHG4 200 mg/kg (Figure 4).

### 3.4. Changes in Absolute Cell Number of Dorsal Skin Tissue and ALN

The absolute cell number of CD19+, CD4+, CD8+, CD4+/CD69+, and CD23+/B220+ for ALN showed a trend of significant increase in DNCB_CTL compared to NC/Nga_Nr (^##^ *p* < 0.01 or ^###^ *p* < 0.001 (compared with NC/Nga_Nr)). In contrast, the absolute cell number of CD19+, CD4+/CD69+, and CD23+/B220+ showed a significant decrease in DNCB_Dexa compared to DNCB_CTL (* *p* < 0.05, ** *p* < 0.01, *** *p* < 0.001 compared with DNCB-CTL). A significant decrease was observed for the absolute cell number of CD19+, CD4+, and CD8+ in DNCB_GU1:DHG1 200 mg/kg and of CD4+, CD8+, and CD4+/CD69+ in DNCB_GU1:DHG2 200 mg/kg. The absolute cell number of CD23+/B220+ also indicated a noticeable decline in DNCB_GU1:DHG4 200 mg/kg (* *p* < 0.05, ** *p* < 0.01, *** *p* < 0.001 compared with DNCB-CTL). The decrease in the absolute cell number across all experimental groups indicated the combined treatment has stronger inhibitory effects compared to the single treatment. For dorsal skin tissue, the absolute cell number of CD4+, CD8+, and Gr-1+/CD11b+ indicated a noticeable rise in DNCB_CTL compared to NC/Nga_Nr (** *p* < 0.01, *** *p* < 0.001 compared with DNCB-CTL). The absolute cell number of CD8+ showed a significant decrease in DNCB_Dexa and across all experimental groups compared to DNCB_CTL (* *p* < 0.05, ** *p* < 0.01, *** *p* < 0.001 compared with DNCB-CTL) (Table 4).

### 3.5. Immunoglobulin E Level in Serum

**Figure 5:**
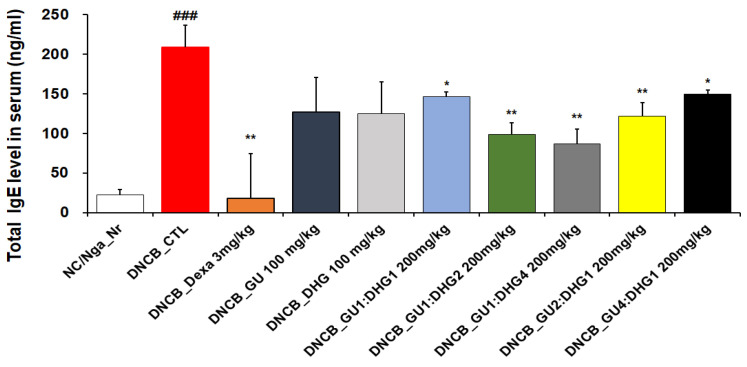
Serum IgE elevation in atopic dermatitis skin lesions induced in NC/Nga mice by DNCB (*n* = 6) ^###^ *p* < 0.001 (compared with NC/Nga_Nr), * *p* < 0.05, ** *p* < 0.01 (compared with DNCB-CTL).

When compared to NC/Nga_ Nr, the amount of serum IgE was considerably higher in DNCB _CTL. As compared to DNCB_ CTL, the IgE level in DNCB_ Dexa was much lower. Across the experimental groups, all groups that received the combined treatment indicated a noticeable decline in IgE level compared to DNCB_CTL, while the most notable reduction in IgE level was observed for DNCB_GU1:DHG4 200 mg/kg, who were administered a high proportion of DHG. A decrease in serum IgE levels indicates that the combination treatment exerts a stronger inhibitory effect compared to a single treatment (Figure 5).

### 3.6. IL-4, IL-5, IL-13, and IFN-γ Protein Production in the Culture Solution of Splenocytes

**Figure 6:**
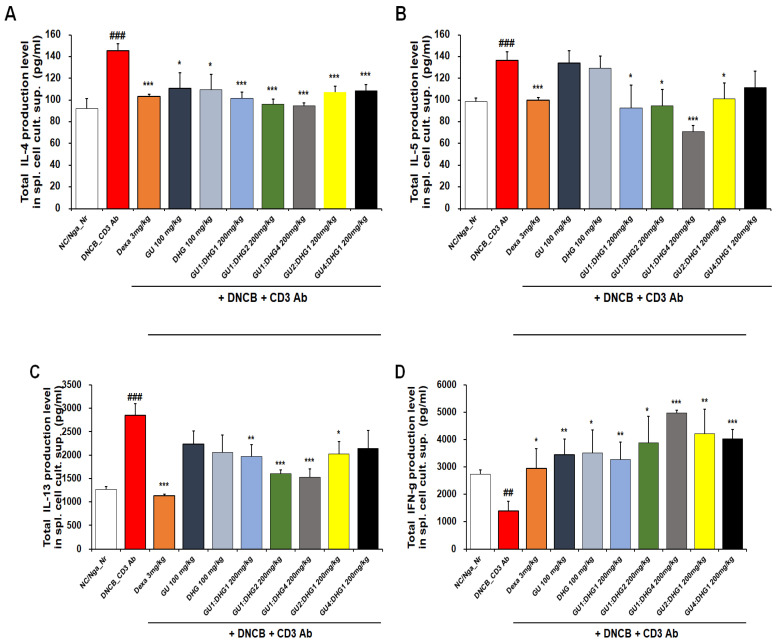
Culture supernatant IL-4, IL-5, IL-13, and IFN-γ levels. Cytokines of splenocytes from NC/Nga mice treated with DNCB (*n* = 6). (**A**) IL-4, (**B**) IL-5, (**C**) IL-13, and (**D**) IFN-γ.^##^ *p* < 0.01, and ^###^ *p* < 0.001 (compared with NC/Nga_Nr), * *p* < 0.05, ** *p* < 0.01, and *** *p* < 0.001 (compared with DNCB-CTL).

The inhibitory effect of reducing serum IL-4, IL-5, IL-13, and IFN-γ protein production in all experimental groups was found to be better with combined treatment than with single treatment. For IL-4, a significant decrease was observed for all groups that received the combined treatment with varying ratios, but a higher rate of decrease was shown by the combined treatment with high DHG content, including DNCB_GU1:DHG1 200 mg/kg, DNCB_GU1:DHG2 200 mg/kg, and DNCB_GU1:DHG4 200 mg/kg (Figure 6A). For IL-5, the highest rate of decrease was shown by DNCB_GU1:DHG4 200 mg/kg (Figure 6B), and for IL-13, higher rates of decrease were shown by the combined treatment with high DHG content, including DNCB_GU1:DHG2 200 mg/kg and DNCB_GU1:DHG4 200 mg/kg (Figure 6C). IFN-γ also showed higher rates of decrease in the combined treatment groups than in the single treatment groups, while DNCB_GU1:DHG4 200 mg/kg exhibited the most significant increase (Figure 6D).

### 3.7. mRNA Expression

**Figure 7:**
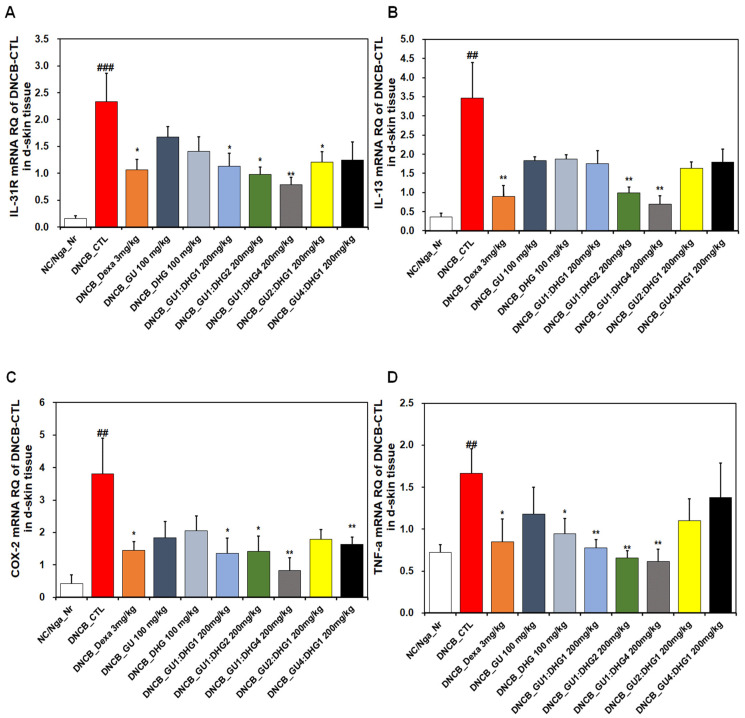
Effects of dexamethasone, GU, DHG, and GU-DHG on IL-31R, IL-13, COX-2, and TNF-α mRNA expression in dorsal skin cells from NC/Nga mice. Total RNA was extracted in dorsal skin tissue. (**A**) IL-31R, (**B**) IL-13, (**C**) COX-2, and (**D**) TNF-α ^##^ *p* < 0.01, and ^###^ *p* < 0.001 (compared with NC/Nga_Nr), * *p* < 0.05, ** *p* < 0.01 (compared with DNCB-CTL).

The analysis of the dorsal skin tissue showed that the relative quantification of the mRNA expression of IL-31R, IL-13, COX-2, and TNF-α, which are genes related to Th2 cells, indicated a noticeable rise in DNCB_CTL compared to NC/Nga_Nr, while significantly decreasing in DNCB_Dexa compared to DNCB_CTL (Figure 7). For IL-31R, a trend of significant decrease was observed for the groups that received combined treatment, compared to DNCB_CTL (Figure 7A), and for IL-13, a significant decrease was observed for DNCB_GU1:DHG2 200 mg/kg, DNCB_GU1:DHG4 200 mg/kg, and DNCB_GU4:DHG1 200 mg/kg (Figure 7B). Likewise, COX-2 and TNF-α showed a significant decrease in the experimental groups that received the treatment containing DHG compared to DNCB_CTL (Figure 7C,D). The highest rate of decrease in the mRNA expression of IL-31R, IL-13, COX-2, and TNF-α was shown by DNCB_GU1:DHG4 200 mg/kg with the highest proportion of DHG.

### 3.8. Histology Analysis of Dorsal Skin Tissue

**Figure 8:**
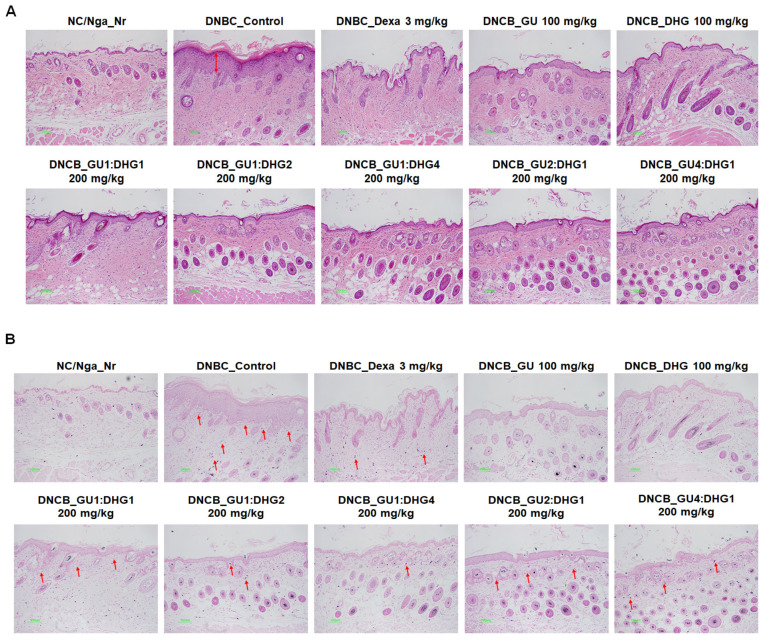
Histological features of the dorsal skin group in NC/Nga mice (*n* = 6). (**A**) NC/Nga mice skin tissues were dyed with hematoxylin and eosin (H&E). (**B**) NC/Nga mice skin tissues were dyed with toluidine blue. The change was visualized using a bright microscope (×200). Mast cell infiltration (red arrow).

Following H&E staining and TB staining, the epidermis in DNCB_CTL exhibited hyperplasia that increased the thickness and caused expansion, while the surrounding tissues showed a significant increase in pigmentation, parakeratosis, adipocyte infiltration, and granulation when compared to NC/Nga_Nr. On the contrary, DNCB_Dexa, DNCB_DHG 100 mg/kg, DNCB_GU1:DHG2 200 mg/kg, and DNCB_GU1:DHG4 200 mg/kg indicated a noticeable rise in the thickness of the epidermis and allergic inflammation in the surrounding tissues after administering the test drug. In DNCB_Dexa and all groups that received the combined treatment, adipocyte infiltration was shown to have significantly decreased (Figure 8).

### 3.9. Immunofluorescence Staining to Evaluate Claudin 1 and Sirt 1 Expression in Dorsal Skin Tissue

**Figure 9:**
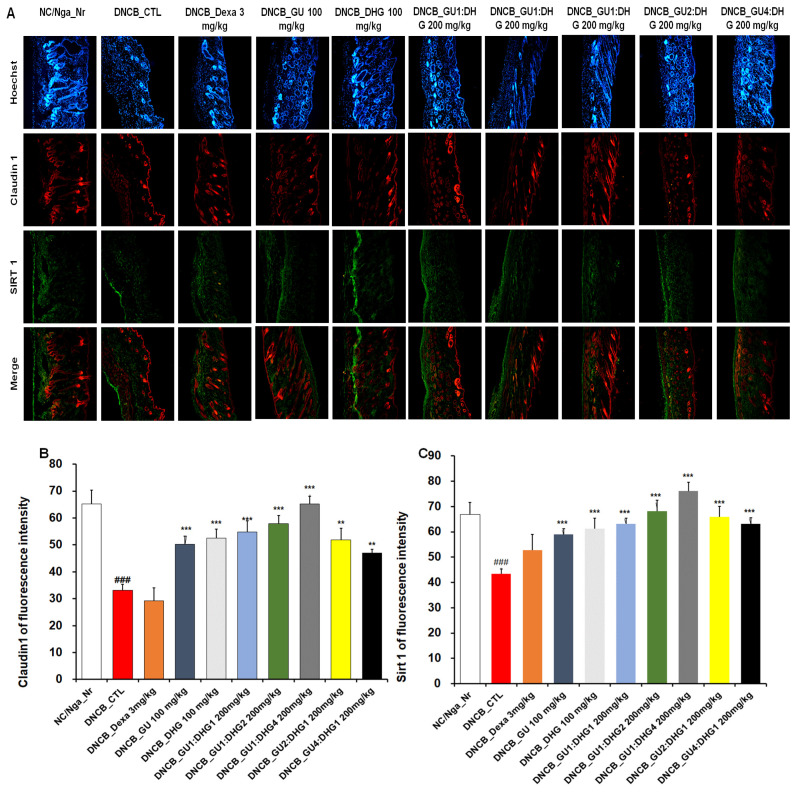
Immunofluorescence staining of dorsal skin tissue. Densitometric quantification of (**B**) Claudin 1 and (**C**) Sirt 1 in mouse dorsal skin tissue in comparison with (**A**) Claudin 1 and Sirt 1 fluorescence staining quantified by the ImageJ program. ^###^ *p* < 0.001 (compared with NC/Nga_Nr),, ** *p* < 0.01, and *** *p* < 0.001 (compared with DNCB-CTL).

The evaluation of Claudin 1 and Sirt 1 expression in dorsal skin tissue showed that the level of expression was reduced in DNCB_CTL compared to NC/Nga_Nr, while in DNCB_Dexa, the skin proteins showed an increasing trend in the level of expression compared to DNCB_CTL, although without statistical significance. Across the experimental groups, a significant increase was found for Claudin 1 expression compared to DNCB_CTL. Sirt 1 expression also showed a significant increase across all experimental groups. The level of increase in Claudin 1 and Sirt 1 expression was more prominent with combined treatment than with either GU or DHG, and the level was higher when the DHG proportion was higher (Figure 9).

### 3.10. Effects of GU, DHG, and GU + DHG Mixtures on the Viability of RAW264.7 Macrophages and HaCaT Keratinocytes

**Figure 10:**
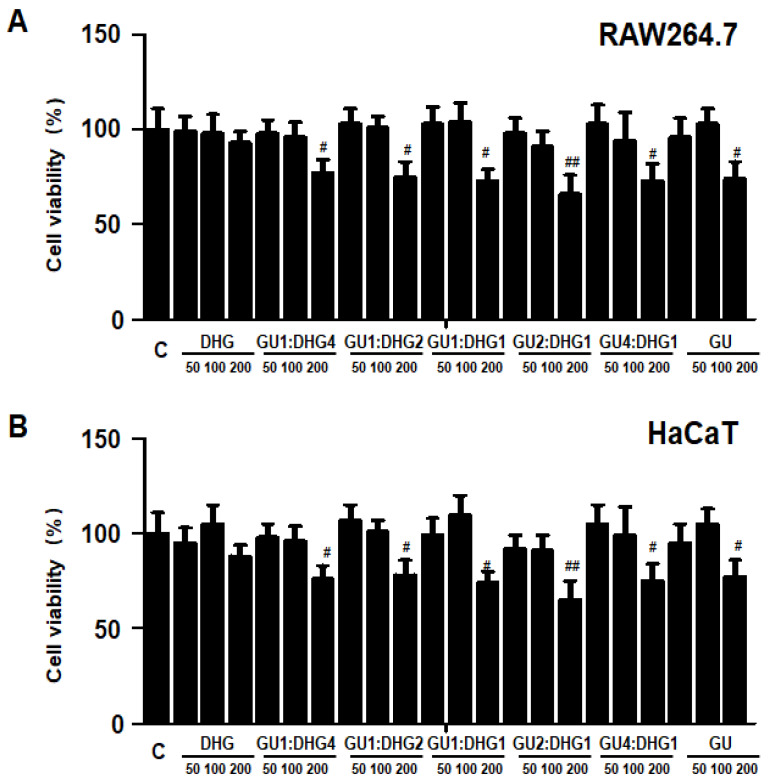
Effects of GU, DHG, and GU + DHG mixtures on the viability of RAW264.7 macrophages (**A**) and HaCaT keratinocytes (**B**). Cytotoxicity was evaluated in cells treated for 24 h with 50 to 200 μg/mL of DHG, GU1:DHG4, GU1:DHG2, GU1:DHG1, GU2:DHG1, GU4:DHG1, and GU extracts. The data are presented as the mean ± SD values of three independent experiments. ^#^ *p* < 0.05, ^##^ *p* < 0.01, vs. control.

The cytotoxicity of DHG, GU, and different ratios of DHG + GU were evaluated using a MTT assay on RAW264.7 cells and HaCaT cells. The cell toxicity of 7 samples was evaluated at various concentration ranges (50–200 µg/mL). Results are shown in Figure 10A,B, DHG 200 μg/mL, GU1:DHG4 100 μg/mL, GU1:DHG2 100 μg/mL, GU1:DHG1 100 μg/mL, GU2:DHG1 100 μg/mL, GU4:DHG1 100 μg/mL and GU 100 μg/mL showed no toxicity on RAW264.7 and HaCaT cells; the following experiments were carried out using these concentrations (Figure 10).

### 3.11. Effects of GU, DHG and GU + DHG Mixture on Inflammatory Mediators Nitrite, PGE_2_, TNF-α, and IL-6 in LPS-Induced RAW264.7 Macrophages

**Figure 11:**
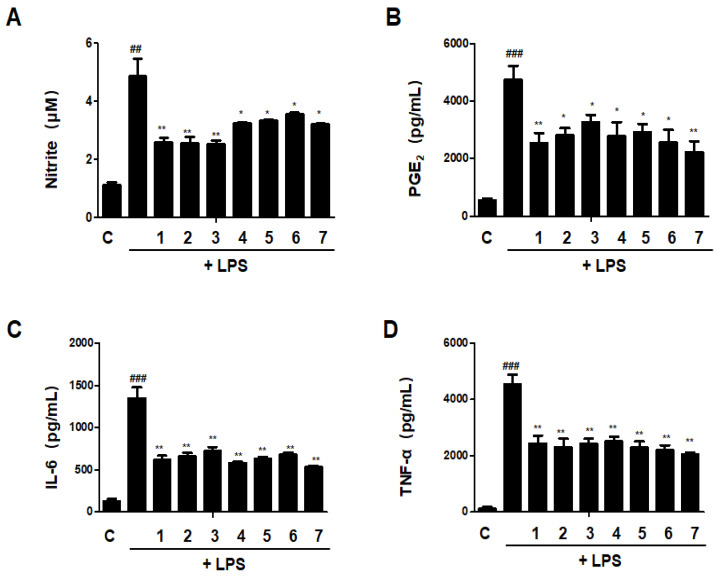
Effects of GU, DHG, and GU + DHG mixtures on inflammatory mediators Nitrite, PGE_2_, TNF-α, and IL-6 in LPS-induced RAW264.7 macrophages (**A**–**D**). Cells are pretreated with DHG at 200 μg/mL (1); GU1:DHG4 at 100 μg/mL (2); GU1:DHG2 at 100 μg/mL (3); GU1:DHG1 at 100 μg/mL (4); GU2:DHG1 at 100 μg/mL (5); GU4:DHG1 at 100 μg/mL (6); and GU at 100 μg/mL (7) for 3 h, then induced with LPS (1 μg/mL) for 24 h. Nitrite, PGE_2_, TNF-α, and IL-6 were measured using the culture supernatant. The data are represented as the mean ± SD of three independent experiments. ^##^ *p* < 0.01, ^###^ *p* < 0.01 vs. control. * *p* < 0.05, ** *p* < 0.01 vs. LPS.

The release of pro-inflammatory mediators is one of the most important functions of macrophages, a differentiated tissue cell type that originated as blood monocytes [18]. We investigated the effects of DHG, GU, and different ratios of DHG + GU on nitrite, PGE_2_, TNF-α, and IL-6 secretion in LPS-induced RAW264.7 macrophages. As shown in Figure 11, LPS treatment significantly increased the secretion. All samples have inhibitory effects on nitrite, PGE_2_, TNF-α, and IL-6 secretion. Especially the groups DHG 200 μg/mL (1), GU1:DHG4 100 μg/mL (2), and GU1:DHG2 100 μg/mL (3) showed significantly reduced nitrite, PGE_2_, TNF-α, and IL-6 secretion.

### 3.12. Effects of GU, DHG, and GU + DHG Mixtures on Protein Expression Levels of Nitric Oxide Synthase (iNOS) and Cyclooxygenase-2 (COX-2) in LPS-Induced RAW264.7 Macrophages

**Figure 12:**
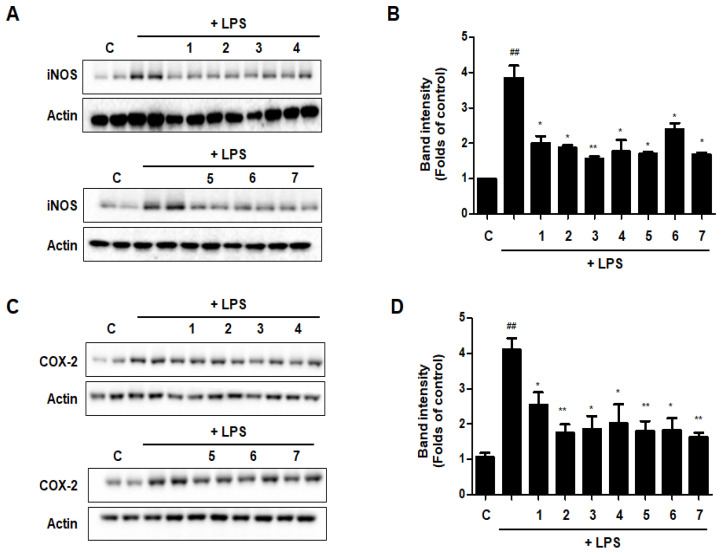
Effects of GU, DHG, and GU + DHG mixtures on protein expression levels of iNOS and COX-2 in LPS-induced RAW264.7 macrophages (**A**–**D**). The data are represented as the mean ± SD of three independent experiments. ^##^ *p* < 0.01, vs. control. * *p* < 0.05 ** *p* < 0.01 vs. LPS.

The expression levels of iNOS and COX-2, which are linked to the production of NO and PGE_2_, were evaluated by western blot analysis to see whether the effects of DHG, GU, and different ratios of DHG + GU involved regulation of the expression of these genes. Results are shown in Figure 12. Seven samples decreased the expression induced by LPS to different degrees. Groups GU1:DHG4 100 μg/mL (2), GU1:DHG2 100 μg/mL (3), and GU 100 μg/mL (7) showed strong inhibitory effects on iNOS and COX-2.

### 3.13. Effects of GU, DHG, and GU + DHG Mixtures on TNF-α/IFN-γ-Induced Pro-Inflammatory Cytokines and Chemokines in HaCaT Cells

**Figure 13:**
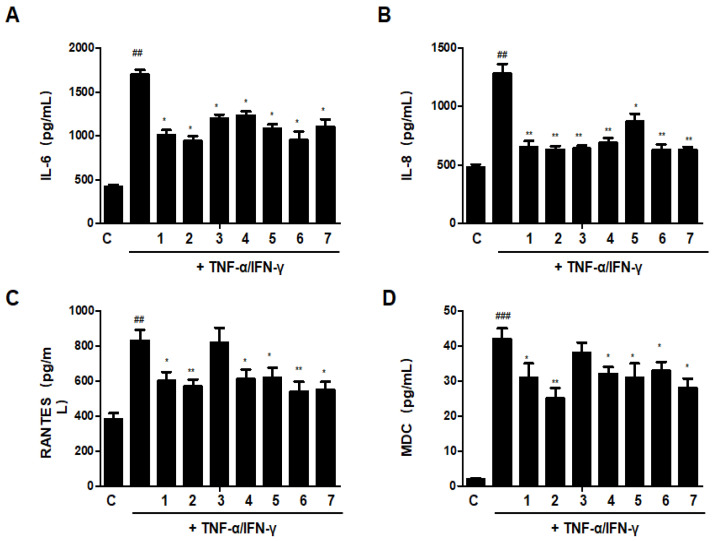
Effects of GU, DHG, and GU + DHG mixtures on TNF-α/IFN-γ-induced pro-inflammatory cytokines and chemokines (**A**–**D**). IL-6, IL-8, RANTES, and MDC were measured using culture supernatant. The data are represented as the mean ± SD of three independent experiments. ^##^ *p* < 0.01, ^###^ *p* < 0.01 vs. control. * *p* < 0.05, ** *p* < 0.01vs. TNF-α/IFN-γ.

The increase of cytokines and chemokines is recognized as a biomarker of chronic inflammation in skin immunological illnesses; as a result, the reduction of these cytokines and chemokines may be essential for the therapy of skin conditions with inflammation [8]. As shown in Figure 13, treatment with TNF-α/IFN-γ could significantly increase the secretion of IL-6, IL-8, RANTES (regulated on activation, normal T cells expressed and secreted), and MDC (macrophage-derived chemokine). Most of the ratios of DHG and GU showed inhibitory effects on IL-6, IL-8, RANTES, and MDC, except group GU1:DHG1 100 μg/mL (3). Among the ratios, group GU1:DHG4 100 μg/mL (2) showed the strongest effects on 4 cytokines and chemokines.

### 3.14. Effects of GU, DHG, and GU + DHG Mixtures on TNF-α/IFN-γ-Induced Expression of ICAM-1 and COX-2 in HaCaT Cells

**Figure 14:**
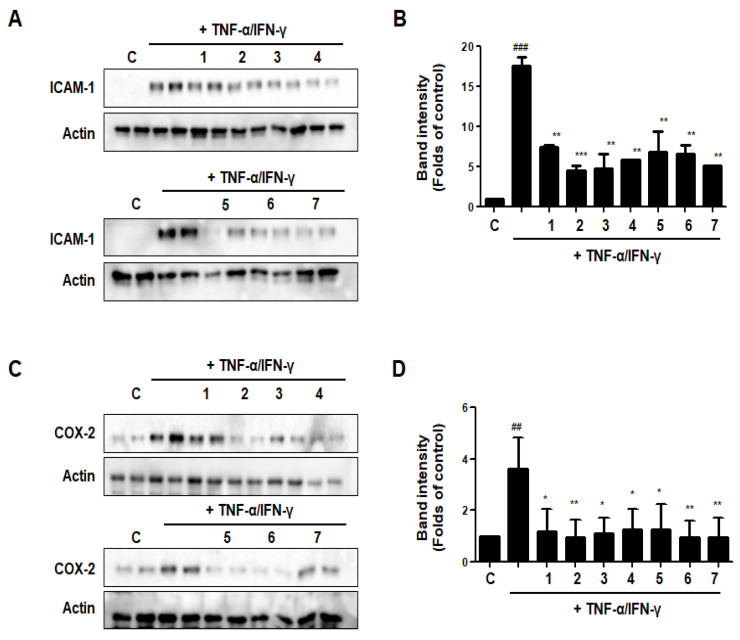
Effects of GU, DHG, and GU + DHG mixtures on TNF-α/IFN-γ-induced expression of ICAM-1 (**A**, **B**), and COX-2 (**C**,**D**) in HaCaT cells. ^##^ *p* < 0.01, ^###^ *p* < 0.01 vs. control. * *p* < 0.05, ** *p* < 0.01 *** *p* < 0.001vs. TNF-α/IFN-γ.

The expression of intercellular adhesion molecule-1 (ICAM-1) rises in response to pro-inflammatory mediators. ICAM-1 expression encourages leukocyte adsorption into adjacent skin tissues. The COX-2 enzyme, which transforms arachidonic acid into prostaglandin H2, is important for skin inflammation. As shown in Figure 14, DHG, GU, and different ratios of DHG + GU all showed strong inhibitory effects on the expression of ICAM-1 and COX-2. The most obvious group is GU1:DHG4 100 μg/mL (2), the expression of ICAM-1 and COX-2 was significantly downregulated.

### 3.15. Effects of GU, DHG, and GU + DHG Mixtures on NF-κB Binding Activity in LPS-Induced RAW264.7 Cells/TNF-α/IFN-γ-Induced HaCaT Cells

**Figure 15:**
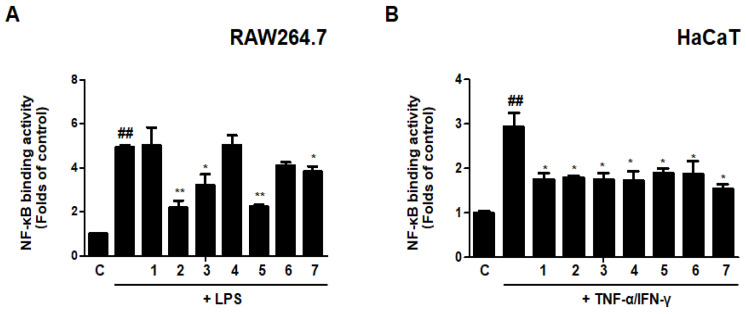
Effects of GU, DHG, and GU + DHG mixtures on NF-κB binding activity in LPS-induced RAW264.7 cells (**A**) and TNF-α/IFN-γ induced HaCaT cells (**B**). Cells are pretreated with DHG 200 μg/mL (1), GU1:DHG4 100 μg/mL (2), GU1:DHG2 100 μg/mL (3), GU1:DHG1 100 μg/mL (4), GU2:DHG1 100 μg/mL (5), GU4:DHG1 100 μg/mL (6), GU 100 μg/mL (7) for 3 h, and then induced TNF-α/IFN-γ (10 μg/mL) or LPS (1 μg/mL) for 15 min. The data are represented as the mean ± SD of three independent experiments. ^##^ *p* < 0.01 vs. control. * *p* < 0.05, ** *p* < 0.01 vs. LPS or TNF-α/IFN-γ.

An important part of innate immunity is NF-κB-mediated inflammation, which appears to be the ultimate common mechanism for the escalation of the inflammatory response to stimuli in immunological disorders of the skin. Therefore, we evaluated the effects of DHG, GU, and different ratios of DHG + GU on NF-κB binding activity using the kit. The results are shown in Figure 15. LPS or TNF-α/IFN-γ could significantly induce the translocation of NF-κB in RAW264.7 and HaCaT cells. In LPS induced RAW264.7 cells, groups GU1:DHG4 100 μg/mL (2) and GU2:DHG1 100 μg/mL (5) showed strong inhibitory effects on NF-κB binding activity. In TNF-α/IFN-γ-induced HaCaT cells, NF-κB binding activity was inhibited by almost all of the extracts. These findings suggested that the NF-B pathway may control the anti-inflammatory effects of DHG, GU, and DHG + GU mixtures.

### 3.16. UHPLC-TOF-HRMS Analysis of the GU1:DHG4 Extract

UHPLC-TOF-HRMS chromatogram analysis of the GU1:DHG4 extract was used to profile ten different chemicals. Using electrospray ionization-MS, the major peaks ascribed to the chromatogram were seen in both positive and negative modes. Additionally, the ion peak and the chemical’s MS data were analyzed in order to identify the compound by comparing them to those found in the literature. The 10 chemicals were comprised of glycosides, alkaloids, flavonoids, isoflavones, and terpenoids, according to the UHPLC-TOF-HRMS analysis (Table 5). According to the results of UHPLC-TOF-HRMS analysis, we could make the distinction that the five compounds, including hydroxyferulic acid, isoliquiritigenin, liquiritin, ononin, and enoxolone, belong to *Glycyrrhiza uralensis* [20]. In addition, the four compounds, including stachydrine, neokestose, 2-[4-[3-[3,4-dihydroxy-4-(hydroxymethyl)oxolan-2-yl]oxy-4,5-dihydroxy-6-(hydroxymethyl)oxan-2-yl]oxyphenyl]-7-hydroxy-2,3-dihydrochromen-4-one, and 7-[3-[(2R,3R,4R)-3,4-dihydroxy-4-(hydroxymethyl)oxolan-2-yl]oxy-4,5-dihydroxy-6-(hydroxymethyl)oxan-2-yl]oxy-3-(4-methoxyphenyl)chromen-4-one may belong to the components of donkey hide gelatin [15]. And sucrose should be a component of both GU and DHG.

## 4. Discussion

Atopic dermatitis generally occurs due to an abnormal increase in the levels of Th2 cytokines such as IL-4, IL-5, and IL-13 [21,22]. In acute atopic dermatitis, biased growth of Th2 cells is observed; however, in the chronic phase, the level of Th1 cytokines shows a simultaneous increase, leading to a highly severe allergic inflammatory response [23]. IFN-γ, as a Th1 cytokine, suppresses the production of IgE and the proliferation of Th2 lymphocytes while upregulating the differentiation and activation of Th1 lymphocytes [24,25]. In the peripheral blood and skin tissue of patients with acute atopic dermatitis, IFN-γ production is inhibited, and as the condition becomes chronic, IFN-γ production increases with decreased expression of Th2 cytokines and production of IgE [26,27,28].

The state of the skin barrier also exerts a substantial influence on the development and maintenance of atopic dermatitis [28]. In a recent study on the relationship between the skin barrier and atopic dermatitis, it was discovered that the tight junction proteins claudin-1 and claudin-23 are less abundant in patients with the condition and that the expression of claudin-1 is negatively correlated with the Th2 immune response [28]. Patients with atopic dermatitis often complain of itchiness in skin lesion areas and may experience a reduced quality of life as the skin barrier damage becomes chronic due to nighttime itching and scratching. Recently, the number of atopic dermatitis patients has steadily increased, and the public’s interest in alternative medicine as a novel therapy has also increased [29].

As a type of alternative medicine, herbal extracts exhibit significant efficacy in a single treatment [30,31,32,33]. In a study analyzing the activity of herbal complexes using structural similarity analysis, many herbal compounds showed enhanced effects in combination with others, and such an increase in activity was shown to be correlated with the metabolic pathways related to amino acids and vitamins [30]. For alternative medicine to be applied in the treatment of atopic dermatitis, the optimum types and mixing ratios of herbal medicine to produce anti-allergic and anti-inflammatory effects should be identified first.

Donkey hide gelatin (DHG) is used in health foods and therapeutic materials. It is obtained via a heat extraction method applied to the hide of *Equus asinus* L., with the main components being gelatin and collagen [15]. DHG has been shown to exhibit antibacterial [29], antioxidant/anti-aging, lung protective [34], and hematopoietic effects [33]. Recently, it has also been reported to have anti-inflammatory and anti-allergic effects through immuno-regulation involving increased Th1 cytokine activity but decreased Th2 cytokine activity [33]. The roots of *Glycyrrhiza uralensis* (GU) are a plant that has been reported to have antioxidant, antidepressant, and anti-hepatitic effects. In addition, glycyrrhetinic acid, one of the main components of GU, is known to exhibit anti-allergic and anti-inflammatory effects through steroid-like effects, including the inhibition of the activation and secretion of cytokines and immune substances. An in vitro study on Jagamcho-tang containing DHG and GU showed an improvement in allergic inflammatory response through the inhibition of Th2 cytokines [35]. With a focus on the fact that GU and DHG among the various components of Jagamcho-tang showed anti-inflammatory and anti-allergic effects, this study aimed to verify the efficacy of each herbal material and to determine the most ideal GU:DHG ratio [36]. According to other previous papers and books, the main components of DHG include protein, amino acids, trace elements, dermatan sulfate, hyaluronic acid, and other polysaccharides and their degradation and binding components [20]. In addition, the main components of GU are triterpenes and flavonoids, as well as alkaloids, polysaccharides, coumarins, amino acids, and trace elements [30]. In our study, we conducted experiments on the components of the GU1:DHG4 extract. As a result of analysis using UHPLC-TOF-HRMS, many compounds were detected, but some of the compounds detected were not commonly seen, and not much research was conducted on them. Therefore, we selected ten compounds in the order of their highest possible contents (Table 3). Additionally, some compounds have also been reported to have biological activity. Stachydrine has demonstrated various bioactivities for the treatment of fibrosis, cardiovascular diseases, cancers, uterine diseases, brain injuries, and inflammation [37]. Isoliquiritigenin may prevent diabetic complications by inhibiting rat lens aldose reductase [38]. Liquiritin acts as an antioxidant and has neuroprotective, anticancer, and anti-inflammatory activities [39]. Therefore, the results on the compounds identified in the UHPLC-TOF-HRMS profiling analysis are meaningful because they are related to the activity of the GU1:DHG4 extract.

The PBMC analysis performed in this study showed that the total cell frequency of CD4+/CD69+ significantly decreased across all experimental groups except DNCB_GU2:DH1 200 mg/kg, compared to DNCB_CTL, while the total cell frequency of Gr-1+/CD11b- also showed a significant decrease in all experimental groups that received the combined treatment with varying ratios of GU and DHG. The absolute cell numbers of CD19+, CD4+, CD8+, CD4+/CD69+, and CD23+/B220+ for ALN were found to have significantly decreased in the experimental groups that received the combined treatment compared to DNCB_CTL. Likewise, the absolute cell number of CD8+ and Gr-1+/CD11b+ in dorsal skin tissue was found to have significantly decreased in most experimental groups. Among the experimental groups, those that received the combined treatment with varying ratios showed more significant changes than those that received the single treatment. The groups that received the combined treatment with a high proportion of DHG showed the highest rates of decrease.

The measured levels of IL-4, IL-5, IL-13, and IFN-γ expression in splenocytes showed that the levels of IL-4, IL-5, and IL-13 expression significantly decreased in the experimental groups compared to DNCB_CTL, whereas the level of IFN-γ expression was found to have significantly increased. The changes in the levels of IL-4, IL-5, and IL-13 in the experimental groups showed an increasing trend in the magnitude of change upon administering the combined treatment compared to the single treatment. In the case of IFN-γ, DNCB_GU1:DHG4 200 mg/kg showed the most significant increase. The mRNA expression levels of IL-31R, IL-13, COX-2, and TNF-α for dorsal skin tissue also showed a significant decrease in the experimental groups that received the combined treatment, and the most prominent decrease was observed for DNCB_GU1:DHG4 200 mg/kg. Thus, it is presumed that the administration of GU and DHG could regulate the production and activation of IFN-γ and a number of immune substances that suppress Th2 cytokines and reduce allergic reactions. The level of serum IgE also showed a significant decrease across all experimental groups that received the combined treatment in varying ratios, compared to DNCB_CTL. The most notable decrease in IgE level was also shown by DNCB_GU1:DHG4 200 mg/kg, which has a high proportion of DHG, and it was found that the rate of decrease was higher upon administration of a combination of test drugs. This implied that the administration of GU and DHG could reduce IgE secretion by preventing allergic reactions mediated by Th2 cytokines. The skin histology analysis also showed significant results for the experimental groups. After toluidine blue staining and H&E staining, the following groups, DNCB_DHG 100 mg/kg, DNCB_GU1:DHG2 200 mg/kg, and DNCB_GU1:DHG4 200 mg/kg, showed a significant fall in inflammatory response after the application of the test drugs. Adipocytes play a crucial role in initiating and maintaining the allergic inflammatory response. DHG and GU are able to reduce the degranulation of adipocytes by regulating the immune substances, which also seem to have an influence on the skin tissue.

The administration of GU and DHG was also found to positively influence the expression of the skin barrier protein. When immune-histofluorescence (IHF) staining was used to measure the expressed levels of Claudin 1 and Sirt 1, all experimental groups showed a significant increase in the expression of Claudin 1 and Sirt 1 compared to DNCB_CTL. The expression of skin proteins in DHG and GU combined treatment also increased more than treatment with either GU or DHG, and the rate of increase showed an increasing trend with the increase in the proportion of DHG. The combination of DHG and GU seems to exert an anti-allergic effect by increasing the expression of skin barrier proteins in the area of damage, whereby the defense function of the skin could be restored. The skin symptom evaluation score across all experimental groups showed a significant decrease. And the fact that the groups that received the combination of GU and DHG treatment showed a higher level of significance for most results in this study. It implies that it may produce better results with the combination of GU and DHG treatment than with each single treatment of GU or DHG. In addition, the experimental groups displayed a significant decrease in the anti-allergic effect based on the ratio between DHG and GU. The GU1:DHG4 ratio is thought to ensure the optimum condition for maximizing the efficacy of the combined treatment.

In LPS-induced RAW264.7 cells, DHG and GU extracts could inhibit the secretion of NO, PGE_2_, IL-6 and TNF-α. And downregulate the expression of iNOS and COX-2. What is more, in TNF-α/IFN-γ induced HaCaT cells, DHG and GU extract also showed strong inhibitory effects on inflammatory cytokines and chemokines, such as IL-6, IL-8, RANTES, and MDC. The expression of ICAM-1 and COX-2 was also decreased. The NF-κB binding activity analysis showed the DHG and GU extracts significantly inhibited the translocation of NF-κB both in RAW 264.7 and HaCaT cells, which may suggest that the DHG and GU extracts could alleviate inflammation by inhibiting the NF-κB pathway. In all in vitro experiments, the GU1:DHG4 group showed the strongest anti-inflammatory effects.

## 5. Conclusions

In this study, we evaluated the inhibitory effects of GU and DHG water extracts on DNCB-induced atopic dermatitis in NC/Nga mice. TNF-α/IFN-γ-treated HaCaT keratinocytes and LPS-stimulated RAW264.7 macrophages. The combined treatment using the water extracts of GU and DHG alleviates atopic dermatitis both in vivo and in vitro. It was found that a DHG:GU ratio of 4:1 showed the best action in the AD models. Results from this study suggested that GU, together with DHG, can be used as the basis for basic research for drug material development in AD disorders. Nevertheless, it is considered that additional research is needed to establish a more detailed formulation ratio of GU and DHG in the future. It will provide a new possibility for the treatment of AD.

## Figures and Tables

**Table 1:** Mice group and treatment.

Group	Concentration
NC/Nga_Nr	
DNCB_CTL	
DNCB_Dexa	3 mg/kg
DNCB_GU	100 mg/kg
DNCB_DHG	100 mg/kg
DNCB_GU1:DHG1	200 mg/kg
DNCB_GU1:DHG2	200 mg/kg
DNCB_GU1:DHG4	200 mg/kg
DNCB_GU2:DHG1	200 mg/kg
DNCB_GU4:DHG1	200 mg/kg

**Table 2:** The sequences of the mouse oligonucleotides.

Gene	Primer	Sequence
GADPH	VIC-probe	5′-CATCCTGCACCACCAACTGCTTAGCC-3′
Il-31R	Forward	5′-ATGCCCAACAAAGCAGAGAC-3′
	Reverse	5′-TGAGAGAACCAGGGAGCTGT-3′
IL-13	Forward	5′-ATGCCCAACAAAGCAGAGAC-3′
	Reverse	5′-TGAGAGAACCAGGGAGCTGT-3′
TNF-α	Forward	5′-TGGGAGGCACTTGCATTGA-3′
	Forward	5′-GGCTTTCCGAATTCACTGGAGCCCT-3′
	Reverse	5′-CCCCGGCCTTCCAAATAAATACATTCATTCATA-3′

**Table 3:** Group and treatment in HaCaT and RAW264.7 cells.

Group Number	Treatment	Concentration
	control	
	TNF-α/IFN-γ or LPS	
1	DHG	200 μg/mL
2	GU1:DHG4	100 μg/mL
3	GU1:DHG2	100 μg/mL
4	GU1:DHG1	100 μg/mL
5	GU2:DHG1	100 μg/mL
6	GU4:DHG1	100 μg/mL
7	GU	100 μg/mL

**Table 4:** The effects of GU, DHG, and GU+DHG mixtures on ALN, dorsal skin tissue, immune cells, and absolute cell number in DNCB-induced atopic dermatitis mice (^##^ *p* < 0.01, and ^###^ *p* < 0.001 compared with NC/Nga_Nr, * *p* < 0.05, ** *p* < 0.01, *** *p* < 0.001 compared with DNCB-CTL).

Cell PHENOTYPES in ALN & D-Skin		DNCB-Induced Atopic Dermatitis Murine Model (Absolute no.)									
		NC/Nga-Nr	DNCB_CTL	DNCB_Dexa. 3 mg/kg	DNCB_GU 100 mg/kg	DNCB_DHG 100 mg/kg	DNCB_GU1:DHG1 200 mg/kg	DNCB_GU1:DHG2 200 mg/kg	DNCB_GU1:DHG4 200 mg/kg	DNCB_GU2:DHG1 200 mg/kg	DNCB_GU4:DHG1 200 mg/kg
CD19+ (×10^5^ cells)	ALN	9.18 ± 6.45	89.78 ± 21.15 ^##^	26.19 ± 7.49 **	61.95 ± 10.95	69.98 ± 5.86	39.49 ± 3.97 *	48.16 ± 1.79	49.12 ± 15.46	78.95 ± 19.06	77.96 ± 34.29
CD4+ (×10^5^ cells)		18.46 ± 13.35	103.37 ± 23.91 ^##^	50.08 ± 15.99 *	72.57 ± 7.63	104.71 ± 3.59	54.84 ± 2.41 *	46.91 ± 13.87 *	66.37 ± 11.46	78.91 ± 14.89	79.60 ± 17.91
CD8+ (×10^5^ cells)		11.6 ± 9.11	105.40± 19.49 ^###^	52.94 ± 21.50 *	58.44 ± 4.85 *	95.49 ± 4.85	62.00 ± 4.76 *	47.07 ± 9.95 **	70.01 ± 13.06	84.78 ± 12.52	86.75 ± 19.60
CD4+/CD69+ (×10^5^ cells)		2.06 ± 1.50	9.20 ± 0.25 ^###^	4.33 ± 1.44 **	7.97 ± 2.24	13.51 ± 2.45	7.89 ± 1.21	5.40 ± 2.33 *	8.40 ± 1.26	9.50 ± 0.41	11.12 ± 2.46
CD23+/B220+ (×10^5^ cells)		10.67 ± 8.12	101.05 ± 35.01 ^##^	23.19 ± 7.21 ***	52.34 ± 8.41 *	79.71 ± 5.61	56.74 ± 4.53 *	56.54 ± 4.45 *	67.40 ± 11.81	79.08 ± 15.07	76.33 ± 30.40
CD4+ (×10^5^ cells)	D-skin	1.02 ± 0.15	14.32 ± 4.56 ^##^	5.16 ± 1.30 *	6.60 ± 0.23	9.70 ± 0.20	7.01 ± 2.23	4.39 ± 0.58 *	2.89 ± 1.13 **	5.96 ± 0.98	5.25 ± 0.74
CD8+ (×10^5^ cells)		0.55 ± 0.04	5.36 ± 0.12 ^###^	0.57 ± 0.25 ***	1.71 ± 0.10 ***	2.21 ± 0.16 ***	1.68 ± 0.62 ***	1.16 ± 0.61 ***	0.58 ± 0.08 ***	2.09 ± 0.61 ***	1.80 ± 0.38 ***
Gr-1+/CD11b+ (×10^5^ cells)		0.37 ± 0.13	3.27 ± 0.73 ^##^	0.96 ± 0.03 **	1.43 ± 0.01 **	1.66 ± 0.30 *	1.65 ± 0.73	1.14 ± 0.01 **	0.52 ± 0.01 **	1.47 ± 1.00	1.68 ± 0.87

**Table 5:** Compounds identified in the GU1:DHG4 extract using ultra-high-performance liquid chromatography-time-of-flight-high-resolution mass spectrometry (UHPLC-TOF-HRMS).

Compound	M	RT (Min)	*m*/*z* Traces (+)	*m*/*z* Traces (–)	MS^2^
Sucrose	342.1	1.30	343.1	–	MS2 (+) [343.1]: 69.4, 85.4, 96.9, 127.6, 145.8, 163.2
Stachydrine	143.1	1.51	144.1	–	MS2 (+) [144.1]: 84.1, 144.4
Hydroxyferulic acid	210.1	8.35	–	209.1	MS2 (–) [209.1]: 59.3, 93.2, 121.1, 165.2
Isoliquiritigenin	256.1	19.27	257.1	–	MS2 (+) [257.1]: 137.1, 147.1, 257.1
Liquiritin	294.2	18.88	–	417.1	MS2 (–) [417.1]: 119.1, 135.1, 255.1
Ononin	430.1	46.22	279.2	–	MS2 (+) [279.2]: 213.1, 237.1, 253.1, 269.1
Enoxolone	470.3	30.36	453.3	–	MS2 (+) [453.3]: 189.1, 235.1, 453.1
Neokestose	504.2	22.89	–	549.2	MS2 (–) [549.2]: 119.4, 135.4, 255.6, 549.2
2-[4-[3-[3,4-dihydroxy-4-(hydroxymethyl)oxolan-2-yl]oxy- 4,5-dihydroxy-6-(hydroxymethyl)oxan-2-yl]oxyphenyl]-7-hydroxy-2,3-dihydrochromen-4-one	550.2	19.37	–	549.2	MS2 (–) [549.2]: 119.2, 135.3, 255.2, 549.2
7-[3-[(2R,3R,4R)-3,4-dihydroxy-4-(hydroxymethyl)oxolan-2-yl]oxy-4,5-dihydroxy-6-(hydroxymethyl)oxan-2-yl]oxy-3-(4-methoxyphenyl)chromen-4-one	562.2	22.51	563.2	–	MS2 (+) [563.2]: 254.1, 269.2

## Data Availability

The data presented in this study are available in this article. Other data supporting the findings of this study are available upon request from the corresponding authors.

## References

[1] Wollenberg A., Barbarot S., Bieber T., Christen-Zaech S., Deleuran M., Fink-Wagner A., Gieler U., Girolomoni G., Lau S., Muraro A. (2018). Consensus-based European guidelines for treatment of atopic eczema (atopic dermatitis) in adults and children. J. Eur. Acad. Dermatol. Venereol..

[2] Nutten S. (2015). Atopic dermatitis: Global epidemiology and risk factors. Ann. Nutr. Metab..

[3] Zhang B., Luo P., Sun J., Li D., Liu Z., Liu X., Zhao Z., Xie X., Yang J., Shen C. (2023). The Epidermal Barrier Structure and Function of Re-Harvested Skin from Non-Scalp Donor Sites. J. Investig. Surg..

[4] Kirschner N., Brandner J.M. (2012). Barriers and more: Functions of tight junction proteins in the skin. Ann. N. Y. Acad. Sci..

[5] Carroll C.L., Balkrishnan R., Feldman S.R., Fleischer A.B., Manuel J.C. (2005). The burden of atopic dermatitis: Impact on the patient, family, and society. Pediatr. Dermatol..

[6] Pescitelli L., Rosi E., Ricceri F., Pimpinelli N., Prignano F. (2021). Novel Therapeutic Approaches and Targets for the Treatment of Atopic Dermatitis. Curr. Pharm. Biotechnol..

[7] Kasraie S.T., Werfel T. (2013). Role of Macrophages in the Pathogenesis of Atopic Dermatitis. Mediat. Inflamm..

[8] Abramovits W. (2005). Atopic dermatitis. J. Am. Acad..

[9] Furue M., Ulzii D., Vu Y.H., Tsuji G., Kido-Nakahara M., Nakahara T. (2019). Pathogenesis of atopic dermatitis: Current paradigm. Iran. J. Immunol..

[10] Dipiro J.T. (2010). Concepts in Clinical Pharmacokinetics.

[11] Reigner D., Williams P., Patel I.H., Steimer J.L., Peck C., van Brummelen P. (1997). An Evaluation of the Integration of Pharmacokinetic and Pharmacodynamic Principles in Clinical Drug Development. Clin. Pharm..

[12] Kim H.U., Ryu J.Y., Lee J.O., Lee S.Y. (2015). A systems approach to traditional oriental medicine. Nat. Biotechnol..

[13] Yin L., Guan E., Zhang Y., Shu Z., Wang B., Wu X., Chen J., Liu J., Fu X., Sun W. (2018). Chemical profile and anti-inflammatory activity of total flavonoids from *Glycyrrhiza uralensis Fisc*. Iran. J. Pharm. Res..

[14] Chen J., Gu X.L., Chen J.G., Luo Y., Wang M.Y., Yang H.Y., Guo X., Zhu X.Q. (2016). Immunomodulatory effects of *Glycyrrhiza uralensis* polysaccharide in glycinin-induced allergic mouse model. Food Agric. Immunol..

[15] Wang D., Ru W., Xu Y., Zhang J., He X., Fan G., Mao B., Zhou X., Qin Y. (2014). Chemical constituents and bioactivities of *Colla corii asini*. Drug Discov. Ther..

[16] Song Y.M., Mao G.N., Kang R.R. (2011). Effect of *Colla corri asini* effervescent granules on immune function in mice. Progr. Vet. Med..

[17] Leung D.Y., Boguniewicz M., Howell M.D., Nomura I., Hamid Q.A. (2004). New insights into atopic dermatitis. J. Clin. Investig..

[18] Suto H., Matsuda H., Mitsuishi K., Hira K., Uchida T., Unno T., Ra C. (1999). NC/Nga mice: A mouse model for atopic dermatitis. Int. Arch Allerg. Immunol..

[19] Lee H., Liu Z.M., Dong L.S., Cheong S.H., Lee D.S. (2022). *Lycopus maackianus* Makino MeOH Extract Exhibits Antioxidant and Anti-Neuroinflammatory Effects in Neuronal Cells and Zebrafish Model. Antioxidants.

[20] Sharifi-Rad J., Quispe C., Herrera-Bravo J., Belén L.H., Kaur R., Kregiel D., Suleria H.A.R. (2021). Glycyrrhiza genus: Enlightening phytochemical components for pharmacological and health-promoting abilities. Oxid. Med. Cell Longev..

[21] Oyoshi M.K., He R., Kumar L., Yoon J., Geha R.S. (2009). Cellular and molecular mechanisms in atopic dermatitis. Adv. Immunol..

[22] Mosmann T.R., Sad S. (1996). The expanding universe of T-cell subsets: Th1, Th2 and more. Immunol. Today.

[23] Grewe M., Bruijnzeel-Koomen C., Schpf E., Thepen T., Langeveld-Wildschut A.G., Ruzicka T., Krutmann J. (1998). A role for Th1 and Th2 cells in the immunopathogenesis of atopic dermatitis. Immunol. Today.

[24] Benedetto A.D., Rafaels N.M., Mcgirt L.Y., Ivanov A.I., Georas S.N., Cheadle C., Berger A.E., Zhang K., Vidyasagar S., Yoshida T. (2010). Tight junction defects in patients with atopic dermatitis. J. Allerg. Clin. Immunol..

[25] Park K.I., Lee M.R., Oh T.W., Kim K.Y., Ma J.Y. (2017). Antibacterial activity and effects of *Colla corii asini* on Salmonella typhimurium invasion in vitro and in vivo. BMC Complement. Altern. Med..

[26] Zhang S., Xu L., Liu Y.X., Fu H.Y., Xiao Z.B., She Y.B. (2018). Characterization of aroma-active components and antioxidant activity analysis of E-jiao (*Colla Corii Asini*) from different geographical origins *Nat*. Prod. Bioprospect..

[27] Wang D., Liu M., Cao J., Cheng Y., Zhuo C., Xu H., Tian S., Zhang Y., Zhang J., Wang F. (2012). 2012. Effect of *Colla corii asini* (E’iao) on D-galactose induced aging mice. Biol. Pharm. Bull..

[28] Tian J., Zhang X., Liu H., Xiang H., Xing J., Zhanga L., Qin X. (2017). The hematinic effect of *Colla corii asini* (Ejiao) using 1H-NMR metabolomics coupled with correlation analysis in APH-induced anemic rats. RSC Adv..

[29] Wang W., Hu X., Zhao Z., Liu P., Hu Y., Zhou J., Zhou D., Wang Z., Guo D., Guo H. (2008). Antidepressant-like effects of liquiritin and isoliquiritin from *Glycyrrhiza uralensis* in the forced swimming test and tail suspension test in mice. Prog. Neuropsychopharmacol. Biol. Psychiatr..

[30] Koan S.W. (2006). Antioxidative Activity of Heat Treated Licorice (Glycyrrhiza uralensis Fisch) Extracts. Korean J. Food Sci. Technol..

[31] Adianti M., Aoki C., Komoto M., Deng L., Shoji I., Wahyuni T.S., Lusida M.I., Soetjipto F.H., Kawahara N., Hotta H. (2014). Anti-hepatitis C virus compounds obtained from *Glycyrrhiza uralensis* and other Glycyrrhizaspecies. Microbiol. Immunol..

[32] Kao T.C., Shyu M.H., Yen G.C. (2010). Glycyrrhizic acid and 18beta-glycyrrhetinic acid inhibit inflammation via PI3K/Akt/GSK3beta signaling and glucocorticoid receptor activation. J. Agric. Food Chem..

[33] Park H.Y., Park S.H., Yoon H.K., Han M.J., Kim D.H. (2004). Anti-allergic activity of 18beta-glycyrrhetinic acid-3-O-beta-D-glucuronide. Arch Pharm. Res..

[34] Hu G., Zhang T.T., Song M.Y. (2021). Effects of *Colla corii asini* on lung function and pathological injury in rats with chronic obstructive pulmonary disease. Basic Clin. Med..

[35] Kang Y.M., Kim H.M., Lee J.S. (2022). *Colla Corii Asini* suppresses the atopic dermatitis-like skin lesions in NC/Nga mice and HaCaT keratinocytes. Pharmacological. Res. Mod. Chin. Med..

[36] Cheng F., Zhou Y., Wang M., Guo C., Cao Z., Zhang R., Peng C. (2020). A review of pharmacological and pharmacokinetic properties of stachydrine. Pharmacol. Res..

[37] Noh H.M., Park S.G., Kim W., Jo E.H., Ki H.H., Kim D.K., Park M.C. (2017). Anti-allergic effects of Jagamcho-tang in ovalbumin-induced atopic dermatitis mouse model. J. Physiol. Pathol. Korean Med..

[38] Lin C.Y., Lin Y.C., Paul C.R., Hsieh D.J.Y., Day C.H., Chen R.J., Huang C.Y. (2022). Isoliquiritigenin ameliorates advanced glycation end-products toxicity on renal proximal tubular epithelial cells. Environ. Toxicol..

[39] Zhou H., Yang T., Lu Z., He X., Quan J., Liu S., Yu L. (2023). Liquiritin exhibits anti-acute lung injury activities through suppressing the JNK/Nur77/c-Jun pathway. Chin. Med..

